# Different routes of insulin administration do not influence serum free thiols in type 1 diabetes mellitus

**DOI:** 10.1002/edm2.88

**Published:** 2019-08-08

**Authors:** Peter R. van Dijk, Femke Waanders, Susan J. J. Logtenberg, Klaas H. Groenier, Titia M. Vriesendorp, Nanne Kleefstra, Harry van Goor, Henk J. G. Bilo

**Affiliations:** ^1^ The Diabetes Centre Isala Zwolle The Netherlands; ^2^ Department of Internal Medicine, University Medical Center University of Groningen Groningen The Netherlands; ^3^ Department of Internal Medicine Isala Zwolle The Netherlands; ^4^ Department of Internal Medicine Diakonessenhuis Utrecht The Netherlands; ^5^ Langerhans Medical Research Group Ommen The Netherlands; ^6^ High & Intensive Care GGZ Drenthe Mental Health Institute Assen The Netherlands; ^7^ Department of Pathology and Medical Biology, University Medical Center University of Groningen Groningen The Netherlands

**Keywords:** insulin, intraperitoneal, redox, subcutaneous, thiols, type 1 diabetes mellitus

## Abstract

**Aims:**

Intraperitoneal (IP) insulin administration is a last‐resort treatment option for selected patients with type 1 diabetes mellitus (T1DM). As the IP route of insulin administration mimics the physiology more closely than the subcutaneous (SC) route, we hypothesized that IP insulin would result in less oxidative stress (expressed as systemic level of free sulphydryl (R‐SH) content) compared to SC insulin in subjects with T1DM.

**Materials and methods:**

Prospective, observational case‐control study. Serum thiol measurements were performed at baseline and at 26 weeks in age‐ and gender‐matched patients with T1DM. Serum‐free thiols, compounds with a R‐SH group that are readily oxidized by reactive oxygen species, are considered to be a marker of systemic redox status.

**Results:**

A total of 176 patients, 39 of which used IP and 141 SC insulin therapy were analysed. Mean baseline R‐SH concentration was 248 (31) μmol/L. In multivariable analysis, the route of insulin therapy had no impact on baseline R‐SH levels. The estimated geometric mean concentrations of R‐SH did not differ significantly between both groups: 264 (95% CI 257, 270) for the IP group and 258 (95% CI 254, 261) for the SC group with a difference of 6 (95% CI −2, 14) μmol/L.

**Conclusions:**

Based on R‐SH as a marker of systemic oxidative stress, these findings demonstrate that the route of insulin administration, IP or SC, does not influence systemic redox status in patients with T1DM.

**Abbreviations**
ALT: alanine aminotransferase
AST: aspartate aminotransferase
BMI: body mass index
CGM: continuous glucose measurement(s)
CIPII: continuous intraperitoneal insulin infusion
CSII: continuous intraperitoneal insulin infusion
CV: coefficient of variation
eGFR: estimated glomerular filtration rate
Gamma‐GT: Gamma‐glutamyl transpeptidase
HbA1c: glycated haemoglobin
HDL: high‐density lipoprotein
IGF: insulin‐like growth factor
IP: intraperitoneal
IQR: interquartile range
LDL: high‐density lipoprotein
MAGE: mean average glucose excursions
MODD: mean of daily differences MDI, multiple daily injections
R‐SH: free sulphydryl
SC: subcutaneous
SD: standard deviation
T1DM: type 1 diabetes mellitus

## 1 INTRODUCTION

Currently, Continuous intraperitoneal insulin infusion (CIPII) is used as a last‐resort treatment option for selected patients with type 1 diabetes mellitus (T1DM) who fail to reach glycaemic control despite intensive subcutaneous (SC) insulin therapy. With CIPII, insulin is infused directly in the intraperitoneal (IP) space resulting in higher concentrations of insulin in the portal vein catchment area, higher hepatic insulin extraction and lower peripheral plasma insulin concentrations compared with SC insulin administration.^1^, ^2^, ^3^

The aberrant production of reactive oxygen species (ROS), due to hyperglycaemia, is considered to be the central element of oxidative stress and, ultimately, plays an important role in the pathogenesis of T1DM, its progression and ultimately micro‐ and macrovascular complications.^4^, ^5^, ^6^, ^7^ Insulin has a pivotal role in the ROS production in T1DM through its effects on glucose‐, IGF1‐, lipid metabolism and its direct impact on the endothelium.^8^ In previous studies, IP administration of insulin resulted in better HbA1c levels, lower glycaemic variability with a lower frequency of hypoglycaemia 9, 10, 11, 12, 13, 14, 15 and (near‐) restoration of insulin‐like growth factor (IGF)‐1 metabolism 16, 17, 18, 19 as compared to SC therapy. Given these effects, it was suggested that not only the insulin level, but also the route of administration might be of importance in the regulation of the redox status in T1DM.^20^, ^21^

Indeed, in the animal model, delivering the same dose of insulin IP resulted in lower hepatic oxidative stress and inflammation as compared to continuous SC insulin delivery.^20^ To date, however, there are no data on the effect of the route of insulin administration on whole‐body redox status in humans. We hypothesized that the route of insulin administration affects the systemic redox status and that the IP route may have a beneficial effect compared with SC insulin therapy. We therefore investigated the effects of CIPII compared with SC insulin administration on redox status in a prospective, observational, matched case‐control study in patients with T1DM.

## 2 MATERIALS AND METHODS

### 2.1 Study design, aims and outcomes

This multicentre study was investigator‐initiated and had a prospective, observational matched case‐control design. Inclusion took place at Isala hospital (Zwolle, the Netherlands) and Diaconessenhuis hospital (Meppel, the Netherlands). Primary aim of this study was to compare the effects of long‐term IP insulin delivery to SC insulin delivery, with respect to glycaemic control. Aim of the present analysis was to test the hypothesis that IP insulin therapy would result in a more favourable redox status compared with SC insulin therapy. Serum‐free thiols, compounds with a free sulphydryl (R‐SH) group that are readily oxidized by reactive oxygen species, were used as marker systemic redox status. From the measures available to measure ROS, we considered R‐SH as an appropriate measure for oxidative stress in the current study: R‐SH are a robust and powerful read‐out of the systemic in vivo reduction‐oxidation (redox) status.^22^ In previous studies, in a variety of diseases, R‐SH have been linked with oxidative stress and clinical outcome.^23^ Secondary outcomes include subanalyses for MDI‐ and CSII‐treated patients and a multivariable regression analysis with baseline R‐SH concentrations as outcome variable.

### 2.2 Patient selection

Cases were subjects on IP insulin therapy using an implantable insulin pump (MIP 2007D, Medtronic/MiniMed) for the past 4 years without interruptions of >30 days, in order to avoid effects related to initiating therapy. Inclusion criteria for cases were have been described in detail previously.^9^ In brief, patients with T1DM, aged 18 to 70 years who fulfilled abovementioned criteria for CIPII and had an HbA1c ≥ 58 mmol/mol and/or ≥5 incidents of hypoglycaemia (defined as glucose < 4.0 mmol/L) per week, were eligible. The SC control group was age‐ and gender‐matched to the cases and consisted of patients with T1DM, using either multiple daily subcutaneous injections (MDI) or continuous subcutaneous insulin infusion (CSII)), for the past 4 years without interruptions of >30 days and a HbA1c at time of matching ≥ 53 mmol/mol. Exclusion criteria for the present study for both cases and controls included the following: impaired renal function (plasma creatinine ≥ 150 µmol/L or Cockcroft‐Gault ≤ 50 mL/min), cardiac problems (unstable angina or myocardial infarction within the previous 12 months or NYHA class III or IV congestive heart failure), cognitive impairment, current or past psychiatric treatment for schizophrenia, cognitive or bipolar disorder, current use of oral corticosteroids or suffering from a condition which necessitated corticosteroids use more than once in the previous 12 months, alcohol or drug abuse, current gravidity or plans to become pregnant during the study.^24^ The ratio of participants on the different therapies (CIPII:MDI:CSII) was 1:2:2.

### 2.3 Study protocol

There were four study visits. During the first visit, baseline characteristics were collected using a standardized case record form. During the second visit (5‐7 days later), laboratory measurements were performed. During the third visit, 26 weeks after visit 1, clinical parameters were collected. During the fourth visit, 5‐7 days after the third visit, laboratory measurements were performed. Throughout the study period, insulin (human insulin of E. Coli origin, 400 IU/mL, trade name: Insuman Implantable^®^, Sanofi‐Aventis) was administered with an implantable pump for IP insulin users and patients using CSII or MDI continued their own insulin regime consisting of fast‐acting insulin analogues and for MDI patients also long‐acting insulin analogues or NPH insulin. All patients received standard care. The implantable insulin pump used during this study and related procedures has been described in more detail previously.^25^, ^26^

### 2.4 Measurements

Demographic and clinical parameters included the following: age, gender, weight, length, blood pressure, smoking and alcohol habits, co‐morbidities, medication use, year of diagnosis of diabetes, presence of microvascular and macrovascular complications and previous insulin therapy (kind of insulin, dosage and, if applicable, the number of daily injections of the previous day). Blood pressure was measured using a blood pressure monitor (M6 comfort; OMRON Healthcare) using the highest mean of 4 measurements (2 on each arm). Patients were instructed to visit the laboratory in a fasting state. Laboratory measurements included creatinine, c‐peptide, total cholesterol, aspartate aminotransferase (AST), alanine aminotransferase (ALT), y‐glutamyl transpeptidase (gamma‐GT), alkaline phosphatase and urine albumin/creatinine ratio and HbA1c. HbA1c was measured with a Primus Ultra2 system using high‐performance liquid chromatography (reference value 20‐42 mmol/mol).

Systemic redox status was assessed using measurements of thiols. Thiols are compounds with a free sulphydryl (R‐SH) moiety. These R‐SH groups are readily oxidized by ROS and other reactive species. The circulating concentrations of total R‐SH have recently been proposed to directly reflect the whole‐body redox status: a decrease in circulating R‐SH concentration represents increased oxidative tone and thus indicates a state of high oxidative stress.^23^, ^27^

Venous blood samples were collected in BD vacutainer^TM^ serum tubes, centrifuged and directly stored in aliquots at −80°C without thawing until measurement of R‐SH. R‐SH were measured as previously described, with minor modifications.^28^, ^29^ Briefly, 75 µL serum was diluted 1:4 in 0.1 mol/L Tris buffer (pH 8.2) and then transferred to a 96‐well plate. Using a Sunrise microplate reader (Tecan Trading AG), background absorption was measured at 412 nm with a reference filter at 630 nm. Subsequently, 10 µL 3.8 mmol/L 5,5′ ‐Dithio‐bis (2‐nitrobenzoic acid) (DTNB; Sigma‐Aldrich) in 0.1 mol/L phosphate buffer (pH 7) was added to the samples. Following 20 minutes of incubation at room temperature, absorption was read again. The concentration of R‐SH in the samples was determined by comparing the absorbance readings to a standard curve of L‐cysteine (15‐1000 µmol/L; Fluka Biochemika, Buchs, Switzerland) in 0.1 mol/L Tris and 10 mmol/L EDTA (pH 8.2).

The 24‐hours interstitial glucose profiles were recorded using a blinded CGM device (iPro2, Medtronic). The CGM device was inserted in the periumbilical area, and in pump users contralateral to the (implanted) insulin pump. Patients were instructed to perform a minimum of 4 blood glucose self‐measurements daily during the CGM period, using a blood glucose metre (Contour XT; Bayer) to calibrate the sensor. All procedures related to the CGM were performed by one, trained physician (PRvD).

### 2.5 Statistical analysis

Results were expressed as mean (with standard deviation (SD)) or median (with interquartile range [IQR]) for normally distributed and non‐normally distributed data, respectively. A significance level of 5% (two‐sided) was used. Normality was examined with Q‐Q plots. Differences between the IP and SC groups averaged over the study period and in time were estimated using the general linear model.

A regression model based on covariate analysis (ANCOVA) was applied in order to adjust for possible baseline imbalances. In the model, the fixed factors CIPII and SC insulin therapy were used as determinants. The difference in scores was determined based on the b‐coefficient of the particular (CIPII or SC) group. Significance of the b‐coefficient was investigated with the Wald test based on a *P* < .05. The quantity of the b‐coefficient, with a 95% CI, gives the difference between both treatment modalities over the study period adjusted for baseline differences.

Furthermore, to evaluate the independent impact of several variables, including the route of insulin administration, on R‐SH concentrations, a multivariate regression model with R‐SH score as primary outcome variable was constructed. For this model, the baseline values were used since the most extensive characterization of the population (eg, including c‐peptide measurements) was performed at baseline. First, univariable linear regression analyses were applied to identify variables that are independently associated with R‐SH. Subsequently, all variables that associated with R‐SH with a *P*‐value of <.1 were included in the multivariable linear regression using backward selection. The quality of the model was described using the accuracy of the prediction by the adjusted *R* ^2^ value. In order to avoid collinearity, only the coefficient of variation (CV) of the CGM measurements was used. The CV measures intraday variation in glucose patterns, is defined as the SD divided by the mean of blood glucose values and is advocated to be the most optimal measure of glycaemic variability.^30^, ^31^, ^32^

Statistical analyses were performed using SPSS (IBM SPSS Statistics for Windows, Version 20.0. , IBM Corp). The study protocol was registered prior to the start of the study (NCT01621308 and NL41037.075.12) and approved by the local medical ethics committee. All patients gave informed consent.

## 3 RESULTS

From December 2012 through August 2013, in total, 335 patients were screened and received information about the study; 190 agreed to participate. After baseline laboratory measurements, 6 patients were excluded because of C‐peptide concentrations exceeding 0.2 nmol/L (n = 4) or an estimated glomerular filtration rate of <40 mL/min/1.73m^2^ (n = 2): 184 patients were followed up during the 26‐week study period. Due to insufficient serum, R‐SH could not be measured in 4 patients. Consequently, 180 patients were included in the present analyses. At baseline, 39 patients were treated with IP insulin and 141 with SC insulin (67 with MDI and 74 CSII). Mean age of the population was 49.8 (12.5) years, diabetes duration 26.1 (12.3) years and HbA1c 63.8 (10.5) mmol/mol (see Table 1).

**Table 1:** Baseline characteristics

	All (n = 180)	IP (n = 39)	SC (n = 141)	MDI (n = 67)	CSII (n = 74)
Clinical					
Male sex (%)	67 (37)	14 (36)	53 (38)	23 (34)	30 (41)
Age (years)	50 (12)	50 (12)	50 (13)	52 (12)	48 (12)
Current smokers (%)	77 (43)	20 (51)	57 (40)	27 (40)	30 (41)
Current alcohol use (%)	58 (32)	10 (26)	48 (34)	24 (36)	24 (32)
BMI (kg/m^2^)	26 (5)	25 (5)	27 (5)	26 (5)	26 (4)
Systolic blood pressure (mm Hg)	137 [123, 148]	136 [126, 152]	133 [123, 147]	134 [123, 150]	133 [123, 145]
Diabetes duration (years)	26 [17, 35]	29 [22, 36]	23 [16, 35]	22 [13, 35]	25 [17, 35]
Retinopathy present (%)	62 (34)	17 (44)	45 (32)	17 (25)	28 (38)
Neuropathy present (%)	50 (28)	20 (51.3)	30 (21)*	16 (24) *	14 (19) *
Nephropathy present (%)	5 (2.8)	2 (5.1)	3 (2.1)	1 (2)	2 (3)
Macrovascular complication present (%)	26 (14)	7 (18)	19 (14)	10 (15)	9 (12)
Basal insulin dose (IU/d/kg)	0.4 [0.2, 0.4]	0.4 [0.3, 0.7]	0.3 [0.2, 0.4]*	0.3 [0.2, 0.4]*	0.3 [0.2, 0.4]*
Bolus insulin dose (IU/d/kg)	0.3 [0.2, 0.4]	0.2 [0.1, 0.3]	0.3 [0.2, 0.4]*	0.4 [0.3, 0.5]*	0.2 [0.2, 0.3] **
Total insulin dose (IU/d/kg)	0.7 [0.5, 0.8]	0.7 [0.5, 0.9]	0.6 [0.5, 0.8]	0.7 [0.5, 0.8]	0.6 [0.4, 0.7]*
Biochemical					
HbA1c (mmol/mol)	63.8 (10.5)	66.9 (14.4)	62.8 (8.9)	62.3 (9.1)	63.4 (8.8)
Fasting glucose (mmol/L)^a^	8.6 (3.7)	8.4 (3.8)	8.7 (3.7)	8.5 (3.8)	8.8 (3.7)
C‐peptide	0.01 [0.01, 0.01]	0.01 [0.01, 0.01]	0.01 [0.01, 0.02]	0.01 [0.01, 0.02]	0.01 [0.01, 0.01]
C‐reactive protein	1.0 [1.0, 3.0]	2.0 [1.0, 5.8]	1.0 [1.0, 3.0]	1.0 [1.0, 3.3]	1.0 [1.0, 2.0]
Creatinine (μmol/L)	69.4 (13.0)	70.0 (12.3)	69.4 (13.2)	69.3 (14.2)	69.4 (12.4)
Albumin (g/L)	41.0 (5.7)	41.8 (6.5)	40.9 (5.5)	40.8 (5.4)	41.0 (5.6)
Alkaline phosphatase (U/L)	73.2 (20.5)	78.1 (18.6)	71.9 (20.8)	72.4 (19.7)	71.4 (21.9)
Gamma‐GT (U/L)	19.0 [14.0, 27.8]	22.0 [14.0, 36.0]	19.0 [14.0, 27.0]	17.0 [13.0, 26.0]	21.0 [14.0, 17.8]
AST (U/L)	23.0 [19.0, 27.0]	24.0 [20.0, 25.0]	23.0 [19.0, 27.0]	23.0 [20.0, 27.0]	23.0 [18.0, 28.3]
ALT (U/L)	18.0 [14.0, 24.8]	20.0 [15.0, 24.0]	18.0 [14.0, 25.0]	18.0 [15.0, 25.0]	18.0 [13.0, 25.0]
Total cholesterol (mmol/L)	4.8 (0.9)	4.9 (1.0)	4.8 (0.8)	4.8 (0.8)	4.7 (0.8)
HDL‐cholesterol	1.8 (0.5)	1.7 (0.5)	1.8 (0.5)	1.8 (0.6)	1.7 (0.4)
LDL‐cholesterol	2.6 (0.8)	2.8 (0.9)	2.6 (0.8)	2.5 (0.8)	2.6 (0.7)
Triglycerides	0.8 [0.6, 1.0]	1.0 [0.7, 1.6]	0.8 [0.6, 1.1]	0.8 [0.7, 1.2]	0.8 [0.6, 1.0]
Microalbuminuria:creatinine ratio	0.9 [0.5, 1.7]	1.2 [0.5, 1.8]	0.8 [0.4, 1.4]	1.0 [0.5, 2.1]	0.8 [0.4, 1.4]
CGM measurements					
Hypoglycaemia (%)	5.4 [1.2, 10.4]	2.4 [0.0, 6.7]	6.1 [1.6, 10.9]	9.7 [3.1, 13.9] *	3.6 [1.0, 7.2] **
Euglycaemia (%)	52.8 [41.6, 62.1]	49.0 [30.9, 59.1]	54.0 [43.7, 62.3]	55.7 [43.0, 61.9]	51.6 [45.0, 62.5]
Hyperglycaemia (%)	40.3 [29.4, 52.2]	46.0 [36.0, 67.4]	38.9 [29.4, 50.6]	36.1 [24.6, 44.3] *	41.7 [31.6, 50.9]**
Mean	9.6 (2.0)	10.6 (2.4)	9.4 (1.8) *	9.0 (1.8) *	9.8 (1.6) **
SD	3.9 (0.9)	3.9 (1.1)	3.8 (0.9)	4.0 (1.0)	3.8 (0.8)
CV	41.0 (9.0)	37.2 (8.4)	41.9 (8.8) *	44.8 (9.6) *	39.3 (7.4) **
MAGE	7.8 (2.5)	7.7 (2.6)	7.9 (2.5)	7.9 (2.7)	7.8 (3.2)
MODD	4.1 (1.3)	3.9 (1.1)	4.1 (1.4)	4.1 (1.7)	4.1 (1.1)

Note

Data are presented as n (%), mean (SD) or median [IQR]. *P*‐values are based on appropriate parametric and nonparametric tests. Retinopathy, neuropathy and nephropathy categories do not add up.

Missing values: MAGE n = 12; MODD n = 13; CV n = 12; fasting glucose n = 22.

Abbreviations: ALT, alanine aminotransferase; AST, aspartate aminotransferase; BMI, body mass index; CSII, continuous intraperitoneal insulin infusion; IP, intraperitoneal; Gamma‐GT, Gamma‐glutamyl transpeptidase; MDI, multiple daily injections; SC, subcutaneous.

* *P* < .05 as compared to CIPII.

** *P* < .05 for MDI versus CSII.

Baseline R‐SH concentrations were normally distributed with a mean concentration of 248 (31) μmol/L (see Appendix S1). According to the multivariate model, factors that had an independent, inverse relation with baseline R‐SH concentrations were age and BMI (see Table 2), whereas fasting glucose and albumin concentrations had a positive relation.

**Table 2:** Univariable and multivariable analyses with R‐SH as outcomes variable

	Univariable, St. Beta	*P*‐value	Multivariable, St. Beta	*P*‐value	Part correlation
Gender (male = 1)	−0.075	.324			
Age (years)	−0.358	<.001	−0.313	<.001	−.302
Current smokers (yes = 1)	0.017	.819			
Current alcohol use (%)	0.094	.219			
BMI (kg/m^2^)	−0.279	<.001	−0.172	.016	−.164
Systolic blood pressure (mm Hg)	−0.107	.159			
Diabetes duration (years)	−0.157	.038			
Retinopathy present (yes = 1)	0.000	.999			
Neuropathy present (yes = 1)	−0.119	.119			
Nephropathy present (yes = 1)	−0.038	.619			
Macrovascular complication present (yes)	−0.122	.110			
Total insulin dose (IU/d/kg)	0.107	.161			
HbA1c (mmol/mol)	0.116	.128			
Fasting glucose (mmol/L)	0.187	.020	0.197	.005	.194
C‐peptide	0.018	.809			
C‐reactive protein	−0.096	.214			
Creatinine (μmol/L)	−2.212	.005			
Albumin (U/L)	0.278	<.001	0.273	<.001	.258
Alkaline phosphatase (U/L)	−0.033	.668			
Gamma‐GT (U/L)	−0.062	.414			
AST (U/L)	0.057	.452			
ALT (U/L)	0.049	.523			
HDL‐cholesterol	−0.033	.669			
LDL‐cholesterol	0.143	.059			
Triglycerides	0.044	.561			
Urine microalbumin:creatinine ratio	−0.124	.105			
MAGE	0.052	.510			
MODD	0.066	.409			
CV	−0.033	.681			
Route of insulin administration (SC = 1)	0.112	.143			

Note

*R* ^2^ for the multivariable model:.345.

Abbreviations: ALT, alanine aminotransferase; AST, aspartate aminotransferase; BMI, body mass index; CV, coefficient of variation; HbA1c, glycated haemoglobin; eGFR, estimated glomerular filtration rate; HDL, high‐density lipoprotein; LDL, high‐density lipoprotein; MAGE, mean average glucose excursions; MODD, mean of daily differences; R‐SH, total free thiol groups; SC, subcutaneous.

The estimated geometric mean concentrations of R‐SH did not differ significantly between both groups: 263.7 (95% CI 257.0, 270.4) for the IP group and 257.7 (95% CI 254.2, 261.3) for the SC group with a difference of 6.0 (95% CI −1.7, 13.5) μmol/L. After adjustment for the total insulin dose, the difference between groups remained nonsignificant: 263.7 (95%CI 256.9, 270.5) for the IP group and 257.7 (95% CI 254.1, 261.3) for the SC group with a difference of 6.0 (95% CI −1.7, 13.8) μmol/L. During the study period, R‐SH concentrations increased among all patients with 20.8 (95% CI 13.2, 28.4) μmol/L (see Table 3). This increase was present in both the IP (18.5 (95% CI 5.1, 31.9) μmol/L) and the SC group (23.1 (95% CI 16.0, 30.3) μmol/L). Concerning the MDI and CSII subgroups, there was also an increase in R‐SH concentrations during the study period and the difference compared with IP insulin was not significant: 8.5 (95% CI −1.9, 18.9) and 3.7 (95% CI − 6.4, 13.9), respectively (see Appendix S2).

**Table 3:** Estimated R‐SH outcomes at baseline and end for all, CIPII‐ and SC‐treated patients

	All			IP			SC			IP vs SC
	Baseline	End	Difference	Baseline	End	Difference	Baseline	End	Difference	Difference
R‐SH (μmol/L)	250.3 (244.8, 255.9)	271.1 (265.9, 276.4)	20.8 (13.2, 28.4)	254.5 (244.6, 264.3)	272.9 (263.7, 282.1)	18.5 (5.1, 31.9)	246.2 (241.0, 251.3)	269.3 (264.3, 274.3)	23.1 (16.0, 30.3)	6.0 (−1.7, 13.5)

Note

Data are presented as estimated mean (95% CI) at baseline and the end of the study period. R‐SH concentrations are in μmol/L.

Abbreviations: IP, intraperitoneal; SC, subcutaneous; R‐SH, total free thiol groups.

## 4 DISCUSSION

The findings in this 26‐week study suggest that the route of insulin administration, IP or SC, does not influence the systemic redox status in patients with T1DM. Although R‐SH concentrations at baseline and at the end of the study were higher among T1DM patients treated with IP insulin, this was not significantly different as compared to the group of patients treated with SC insulin.

In the only previous study (by Dal et al) that investigated the influence of the route of insulin administration on the redox status, an identical dose of insulin was administered for 4 weeks via the IP and SC route to STZ‐induced diabetic rodents.^20^ This resulted in less liver and global inflammation, as measured by alpha‐2‐macroglobulin with IP insulin.^20^ In addition, they observed increases in IGF‐1 and a decrease in blood glucose concentration.

The previous observations that IP insulin administration in human T1DM patients results in lower Hba1c, less glycaemic variability and higher IGF‐1 levels as compared to SC insulin treatment 19, 24, 33 led to the hypothesis tested in this study that IP insulin therapy per se would have beneficial effect on the redox status as compared to SC insulin. However, no differences in redox status between the different routes of insulin administration were observed in the current study.

This may suggest that the pathways (ie, glycaemia and IGF‐1 metabolism) that influence redox status and are known to be differently influenced by IP and SC insulin, counteract each other resulting in a stable redox status. Despite the fact that no differences in lipid metabolism and high‐sensitive CRP concentrations were present in the present study, it cannot be ruled out that these or other (unmeasured) pathways influenced redox status in the present study.

Obviously, differences in treatment duration, species and the parameters of oxidative stress used could also account for the different findings of this study as compared to Dal et al In the current study, patients were stable on their mode of therapy for at least 4 years while in the study of Dahl et al rats were treated for 4 weeks.^20^ In contrast to the study of Dahl et al in which alpha‐2‐macroglobulin was used as a marker of systemic oxidative stress, R‐SH concentrations were used in the current study. Unfortunately, to the best of our knowledge, no direct comparisons between alpha‐2‐macroglobulin and R‐SH as marker of systemic oxidative stress are available.

As expected, obesity and ageing were inversely associated with increased oxidative stress.^34^ To the best of our knowledge, this is the first study among persons with T1DM to demonstrate that age could be a more dominant determinant of oxidative stress than glycaemia. Although several limitations of this study (as mentioned below) should be considered here, this interesting finding needs confirmation. At baseline, R‐SH was also associated with albumin and fasting glucose. In serum, the concentration of all thiols added together is lower than that intracellular, with albumin being the most abundant thiol.^35^ Therefore, the baseline correlation association of R‐SH and albumin was more or less expected. Ates et al were the first to investigate levels of free thiols in persons with T1DM. In their study, among 38 subjects significantly more thiol oxidation among patients with T1DM was present as compared to healthy controls.^36^ In addition, a correlation between R‐SH with glucose, HbA1c and inflammatory markers was observed. The results of the present study only partly support these findings by finding significant associations of R‐SH with fasting glucose. In addition, thiol concentrations were considerably lower in the present study: 248 vs 336 μmol/L in the study by Ates et al Differences in size and characteristics of the study population may be held accountable for the discrepancies: in the current study, there were more subjects that were older (50 vs 30 years). On the other hand, patients had lower HbA1c (64 vs 88 mmol/mol), lower glucose concentration (8.6 vs 11.4) and a lower grade of inflammation measured as CRP (1.0 vs 3.4) as compared to the study by Ates et al.^36^ Taken together, this may indicate that age is a more important determinant of thiol concentrations than glycaemia.

Strengths of the present study include the inclusion of patients who have been using their current route of therapy for at least 4 years, thus creating a stable situation, and measurements made on two points in time. During the study period, there was an increase of R‐SH concentrations in both treatment groups. To explore this increase in more detail, we post hoc repeated this analysis for R‐SH using total insulin dose, glucose or albumin as covariates. However, the R‐SH increase over the study period remained significant. This may indicate that other nonmeasured variables (eg, diet or exercise) were involved here. Other limitations should be mentioned. Major limitation of the present study is the nonrandomized design. Ideally, R‐SH measurements should be performed prior and after initiation of IP insulin therapy to compare the changes in R‐SH status from baseline (with SC insulin therapy). However, the global shortage of implantable insulin pumps precludes such a study design. Taken together, no conclusions can be made regarding causality of our findings. And although we prespecified redox status as a secondary outcome in the study protocol, no separate power calculation was performed to detect potential relevant differences in R‐SH. By using the directions of the 95% confidence intervals, one could hypothesize that there are undetected differences in R‐SH between the IP and SC group. Finally, the lack of information with regard to other plasma antioxidant species such as ascorbate, uric acid and small‐molecular‐weight thiols and markers of inflammation (due to cost constraints) should be mentioned.

In conclusion, the findings in this study demonstrate that the route of insulin administration, IP or SC, does not influence systemic redox status in subjects with T1DM, at least, measured as per R‐SH group detection.

## CONFLICT OF INTEREST

The authors declare that they have no financial or other relationships that might lead to a conflict of interest. PRv.D. is the guarantor of this work and, as such, had full access to all the data in the study and takes responsibility for the integrity of the data and the accuracy of the data analysis.

## AUTHORS’ CONTRIBUTIONS

PRv.D. involved in design, inclusion of patients, measurements, statistical analysis and writing manuscript. KG involved in design, statistical analysis and critically reviewing manuscript. TV involved in critically reviewing manuscript. H.v.G. involved in design and measurements. NK, FW, SJJL and HJGB involved in design and critically reviewing manuscript.

## ETHICAL APPROVAL

The study protocol was in compliance with the ethical standards laid down in the 1964 Declaration of Helsinki and its later amendments. The study protocol was registered prior to the start of the study (NCT01621308 and NL41037.075.12) and approved by the local medical ethics committee. All patients gave informed consent.

## Data Availability

Data will be made available by the authors upon (reasonable) request.
