# Supplementary material for: Different routes of insulin administration do not influence serum free thiols in type 1 diabetes mellitus
Source: Endocrinol Diabetes Metab. 2019 Aug 8;2(4):e00088. doi: 10.1002/edm2.88 (PMC6775445; doi:10.1002/edm2.88)
Supplement: Supplementary file 1 [file EDM2-2-e00088-s001.docx]

**Appendix 1.** Histogram and detrended q-q plots of R-SH concentrations in T1DM


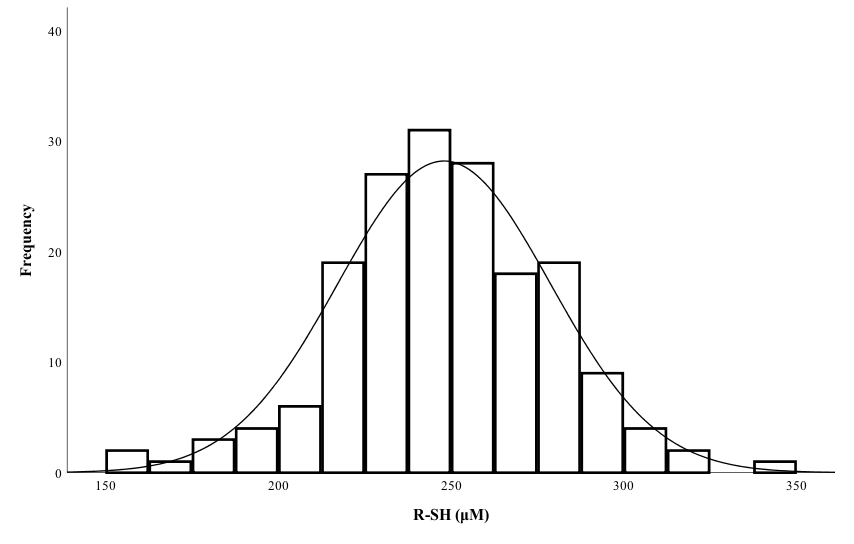


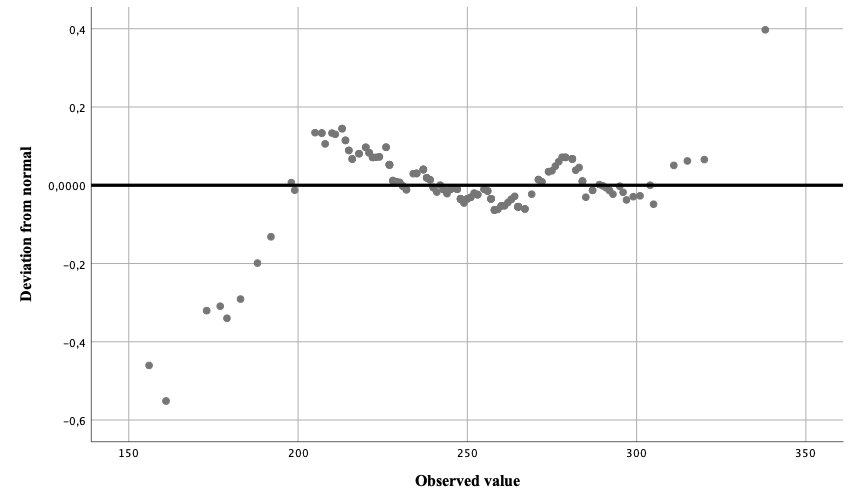


**Appendix 2.** Estimated R-SH outcomes at baseline and end for MDI and CSII treated patients.

|  | **MDI**  Baseline | End | Difference | **IP vs. MDI**  Baseline | **CSII**  Baseline | End | Difference | **IP vs. CSII**  Difference |
| --- | --- | --- | --- | --- | --- | --- | --- | --- |
| R-SH (μM) | 241.8 (234.1, 249.5) | 268.5  (261.3, 275.7) | 26.7  (16.3, 37.2) | 8.5  (-1.9, 18.9) | 249.8 (242.8, 256.8) | 270.1 (263.1, 277.0) | 20.2  (10.4, 30.1) | 3.7  (-6.4, 13.9) |

Data are presented as estimated mean (95% CI) at baseline and the end of the study period. R-SH concentrations are in μM. Abbreviations: IP, intraperitoneal; MDI, multiple daily injections; CSII, continuous subcutaneous insulin infusion.

**Appendix 3.**

|  | **All**  Baseline | End | Difference | **IP**  Baseline | End | Difference | **SC**  Baseline | End | Difference | **IP vs. SC**  Difference |
| --- | --- | --- | --- | --- | --- | --- | --- | --- | --- | --- |
| R-SH (μM) | 250.3 (244.8, 255.9) | 271.1 (265.9, 276.4) | 20.8  (13.2, 28.4) * | 254.5  (244.6, 264.3) | 272.9 (263.7, 282.1) | 18.5  (5.1, 31.9) | 246.2  (241.0, 251.3) | 269.3 (264.3, 274.3) | 23.1  (16.0, 30.3) | 6.0  (-1.7, 13.5) |
| R-SH (μM per gram albumin) | 6.2  (6.0, 6.4) | 6.8  (.5, 7.2) | 0.66  (0.27, 1.1)* | 6.2  (5.8, 6.6) | 6.7  (6.1, 7.3) | 0.6  (-0.23, 1.2) | 6.1  (5.9, 6.3) | 7.0  (6.6, 7.3) | 0.9 (0.5, 1.2) * | -0.1 (-0.5, 0.3) |

|  | **MDI**  Baseline | End | Difference | **IP vs. MDI**  Baseline | **CSII**  Baseline | End | Difference | **IP vs. CSII**  Difference |
| --- | --- | --- | --- | --- | --- | --- | --- | --- |
| R-SH (μM) | 241.8  (234.1, 249.5) | 268.5  (261.3, 275.7) | 26.7  (16.3, 37.2) | 8.5  (-1.9, 18.9) | 249.8  (242.8, 256.8) | 270.1  (263.1, 277.0) | 20.2  (10.4, 30.1) | 3.7  (-6.4, 13.9) |
| R-SH (μM per gram albumin) | 6.0  (5.8, 6.3) | 6.9  (6.4, 7.3) | 0.8  (0.3, 1.4)* | 0.01  (-0.5 0.5) | 6.2  (5.8, 6.6) | 7.1  (6.6, 7.5) | 0.9  (0.4 1.4) | -0.18  (-0.71, 0.35) |
